# Effects of Sodium Monensin and a Tannin–Yeast Blend on Intake, Milk Yield, and Methane Emissions in Lactating Holstein Cows

**DOI:** 10.3390/ani16091345

**Published:** 2026-04-28

**Authors:** Letícia Guerra Piuzana, Thierry Ribeiro Tomich, Polyana Pizzi Rotta, Daiane Carvalho, Wellington Paulo Fernandes Amorim, Luis Henrique Rodrigues Silva, Jaimison Vinícius Ferreira Vieira, Emília Ferreira Ribeiro, Alex Lopes da Silva

**Affiliations:** 1 Departament of Animal Science, Universidade Federal de Viçosa, Viçosa 36570-900, MG, Brazil; leticiaguerrapiuzana@gmail.com (L.G.P.); polyana.rotta@ufv.br (P.P.R.); wellington.amorim@ufv.br (W.P.F.A.); luis.henrique@ufv.br (L.H.R.S.); jaimison.vieira@ufv.br (J.V.F.V.); emilia.ribeiro@ufv.br (E.F.R.); 2 Embrapa Dairy Cattle, Juiz de Fora 36038-330, MG, Brazil; thierry.tomich@embrapa.br; 3 American Nutrients of Brazil, Teutonia 95890-000, RS, Brazil; ped@americannutrients.com.br

**Keywords:** additives, methane, monensin, tannins

## Abstract

This study evaluated the effects of sodium monensin or a blend containing condensed tannins and yeast products on intake, digestibility, performance, and methane emissions in lactating Holstein cows. Nine cows (three rumen-fistulated and six non-fistulated) were assigned to three 3 × 3 Latin squares. The treatments were: a control (CON), sodium monensin (MON; 12 mg/kg of dry matter [DM]), condensed *Acacia* tannins and *Saccharomyces cerevisiae* yeast blend (SUP; 2 g/kg of DM). The trial lasted 84 days, with three 28-day periods. Neutral detergent fiber (NDF) intake was higher in CON and SUP (*p* = 0.029). Milk yield, energy-corrected milk, and milk composition did not differ (*p* > 0.05). The total methane emissions were not affected by treatments (*p* > 0.05). Methane yield/Kg of DM intake (DMI), organic matter intake (OMI), and digestible OM tended to be lower in SUP (*p* = 0.091, *p* = 0.093, *p* = 0.086). SUP increased the DM, crude protein (CP), and NDF ingestion rates (*p* = 0.049, *p* = 0.028, *p* = 0.013) and decreased the CP rumen pool (*p* = 0.014). Rumen pH tended to be higher in SUP (*p* = 0.067). The potentially digestible NDF digestion rate decreased in MON (*p* = 0.007). Finally, SUP-treated animals showed a tendency to reduce their methane yield relative to DMI, OMI, and digestible OM. Further studies should investigate the long-term impacts of supplementation, rumen microbiome changes, and underlying mechanisms driving methane mitigation.

## Simple Summary

The global demand for milk continues to increase, but so do concerns about the environmental impact of dairy farming, particularly methane, a potent greenhouse gas produced by cows that contributes to climate change. In this study with Holstein cows, three diets were tested: a control diet (no additive), one with sodium monensin, and one with tannins and yeast products. The objective was to evaluate whether these supplements could improve cow efficiency and reduce methane emissions. The results showed that the diet containing tannins and yeast products reduced the methane yield relative to feed intake. These findings suggest that the use of tannins and yeast products is a promising strategy to make milk production more sustainable, although further research is needed to clarify the underlying mechanisms and their effects on the rumen microbiota.

## 1. Introduction

The demand for animal products, especially milk, is increasing. It is estimated that food production will need to increase by 58% by 2050 compared to 2010 [1], which will require intensification and improvement of dairy production systems. However, in addition to the increasing demand for food, the need to reduce the environmental impact caused by these systems has also gained increasing importance in recent decades [2]. Therefore, dairy production must strive for efficiency and environmental sustainability [3,4].

At present, climate change caused by increased greenhouse gas emissions has attracted a great deal of attention in the global debate. These changes, especially global warming, are caused by the high concentration of mainly carbon dioxide (CO_2_), nitrous oxide (N_2_O), and methane (CH_4_) in the atmosphere [5]. The CH_4_ is the second most important greenhouse gas, with a global warming potential that is 28 times greater than that of CO_2_ over a 100-year period [6]. Nevertheless, its atmospheric lifetime is 8.6 years, while CO_2_ is 120 years [7].

According to Climate Watch [6], agriculture was responsible for the emission of 5.87 gigatons (Gt) of greenhouse gases in 2020, which corresponds to 12.35% of total emissions. Most of these emissions from agriculture are attributed to CH_4_, which is responsible for 3.54 Gt of CO_2_ equivalents, accounting for 60% of total emissions. According to the same platform, enteric fermentation accounted for the emission of 2.8 Gt of CO_2_ equivalents in 2019. This scenario explains the global focus on CH_4_ mitigation, which aims to reduce the impact caused by the emission of CH_4_ in agriculture.

The breakdown of dietary carbohydrates in the rumen is responsible for the production of volatile fatty acids (VFA), CO_2_ and H_2_. Hydrogen is present in the rumen in two forms: gaseous H_2_ and dissolved H_2_, which is available for utilization by microorganisms. Typically, H_2_ does not accumulate in the rumen because it is rapidly used by methanogenic archaea to produce CH_4_. This process is crucial, as the accumulation of H_2_ in the rumen could limit rumen fermentation [1,8].

Emissions from ruminants represent a major environmental concern due to their contribution to global greenhouse gas emissions and climate change [5,7]. Reducing enteric CH_4_ is therefore a priority in efforts to improve the sustainability of dairy production systems.

Beyond environmental concerns, energy losses due to methanogenesis may account for 2–12% of the gross energy intake in ruminants, energy that could otherwise be used for physiological processes [5,8,9]. This energetic inefficiency underscores the need for nutritional strategies aimed at mitigating CH_4_ emissions in dairy production systems while improving feed utilization, including the use of feed additives.

The main feed additive used in animal husbandry is sodium monensin, a carboxylic polyether produced by the fermentation of the bacterium *Streptomyces cinnamonensis*, which is classified as antimicrobial and can indirectly contribute to CH_4_ mitigation [10,11]. Monensin inhibits Gram-positive bacteria due to its ability to bind the lipid bilayer of the cell membrane, translocate protons (H+) and ions across the membrane, leading to cell death and stimulating the proliferation of Gram-negative bacteria [12]. According to the results of recent meta-analyses, the average reduction in CH_4_ emissions is around 5%, as reported by Marumo et al. [13], and ranges from 4 to 10%, as stated by Ahvanooei et al. [14]. However, the European Union has banned the use of antimicrobials for non-therapeutic purposes since 2006 [15]. In light of this situation, research into alternative feed additives such as tannins and yeast compounds is increasing [16,17,18,19].

Tannins are described as polyphenolic plant compounds with a complex structure and affinity for proteins and other compounds, which can be classified as condensed or hydrolyzable tannins [1,20]. Polyphenols are bioactive components that have a strong ability to scavenge free radicals, acting as antioxidants and improving health, gut development and other production traits in different types of animals [21].

The mechanism of action of tannins in CH_4_ reduction is not yet well understood, but it is likely that these factors are associated with a reduction in fiber digestibility and, consequently, a reduction in H_2_ formation. This reduction may occur through the direct inhibition of methanogenic archaea or indirectly by affecting protozoa that have a symbiotic relationship with archaea [8,22,23]. In addition, tannins can form a complex with proteins and carbohydrates that makes them inaccessible for degradation in the rumen, resulting in reduced nutrient availability for the growth of methanogenic archaea [24].

Yeast is a probiotic used to improve feed efficiency and rumen fermentation, by stabilizing ruminal pH and stimulating the proliferation and activity of fiber-digesting microorganisms [25]. Live yeast and yeast-derived products promote a more favorable rumen environment by scavenging oxygen, stimulating the growth of anaerobic cellulolytic microorganisms, and increasing the population and activity of key fibrolytic species such as *Ruminococcus* spp. and *Fibrobacter succinogenes*. These effects contribute to improved fiber degradation, volatile fatty acid production, and overall feed utilization.

Yeast products include live yeast, yeast cultures, yeast cell wall and purified cell wall components such as mannan-oligosaccharides and β-glucans [26]. These yeast-derived cell wall compounds can bind tannins and other anti-nutritional factors, reducing their inhibitory effects on rumen microbes and digestive enzymes, helping to preserve fermentation efficiency in diets that contain tannins.

The aim of this study was to investigate the effects of a blend of tannins and yeast products or sodium monensin on intake, digestibility, productive performance, and CH_4_ emissions in lactating Holstein cows. We hypothesized that the combination of tannins and yeast products would exert complementary effects on rumen fermentation, leading to reduced CH_4_ emissions while maintaining or improving productive performance compared to sodium monensin.

## 2. Materials and Methods

All procedures involving the use of animals were reviewed and approved by the Ethics Committee for the Use of Production Animals at the Department of Animal Science of the Universidade Federal de Viçosa, Viçosa, Minas Gerais, Brazil, under protocol number 020/2022, in advance. 

### 2.1. Animals, Treatments, and Management 

Nine Holstein cows were used in the study, including three primiparous and six multiparous animals (three rumen-fistulated and six non-fistulated). The animals presented an average of 37.3 ± 4.1 kg of milk yield, 115 ± 42 days in milk, and 603 ± 68 kg of body weight.

The experiment followed a design with three grouped 3 × 3 Latin squares. Each Latin square consisted of three cows and three experimental periods, allowing each cow to receive all three dietary treatments over the course of the experiment. Each animal served as its own control, thereby reducing between-animal variability and increasing statistical efficiency.

The animals were subjected to 3 treatments: control (CON), monensin (MON; Rumensin^®^, Elanco Animal Health, Greenfield, IN, USA) and a supplement based on *Acacia* tannins and *Saccharomyces cerevisiae* yeast products (SUP). In the MON treatment, the animals received 12 mg/kg dry matter (DM) of sodium monensin, while in the SUP treatment, the animals received 2 g/kg DM of tannins and yeast products as an additive. The supplement was included as a strategy to modulate rumen fermentation, aiming to improve nutrient utilization and reduce methane production. In all treatments, virginiamycin was included in the basal diet and provided equally across treatments. Therefore, any potential effects of virginiamycin on rumen fermentation or methane production would be consistent among treatments and would not affect the comparative evaluation of the dietary additives.

The animals were housed in a tie-stall system, with 12.7 m^2^ available for each animal. The stalls were equipped with a positive pressure ventilation system and individual feeders and watering systems. The diet offered is described in Table 1, with a forage to concentrate ratio of 60:40 on a DM basis. Diets were formulated for a milk yield of 38 kg/day and offered at 07:00, 15:00, and 21:00, allowing ad libitum intake, with approximately 5% orts (as fed) permitted daily. The animals were milked three times a day, at 06:30, 14:30, and 20:30.

### 2.2. Experimental Period, Sample Collection, and Laboratory Analysis 

The experimental period lasted 84 days, divided into three periods of 28 days each, whereby the animals had 14 days to adapt to the diet. From the 15th to the 17th day of each trial period, the milk yield of the animals was measured by mechanical milking with an electronic flow meter. During this period, milk samples were collected at all milkings and individually analyzed for fat, protein, lactose and total solids content, using the Lactoscan S_LP ultrasonic milk analyzer (Milkotronic Ltd., Nova Zagora, Bulgaria). The average values obtained were then calculated and composed according to milk production. In addition, a composite sample representing the milk from all three milkings on the last day of each collection period was sent to a laboratory for analysis of the somatic cell count (SCC) by flow cytometry, and casein and milk urea nitrogen (MUN) by infrared analysis. Finally, energy-corrected milk (ECM; kg/d) was calculated as suggested by NASEM [27] as follows:

ECM = 0.252 × kg Milk + 12.30 × kg Fat + 7.77 × kg Protein (1)

Regarding feed sample analysis, during the 15th to the 17th day of each trial period, samples of orts, silage, and hay were collected daily, weighed, and composited per animal across the three days. The composite samples were then dried for 72 h at 55 °C in a forced-air oven and grounded to 2 and 1 mm. Samples grounded to 1 mm were used for the determination of dry matter (DM; method G-003/1), organic matter (OM; method M-001/2), crude protein (CP; Kjeldahl method N-001/2), and neutral detergent fiber (NDF; method F-002/2) according to Detmann et al. [28]. Samples grounded to 2 mm were used for the determination of indigestible neutral detergent fiber (iNDF) (method F-009/2) [28]. The intake of the animals was determined based on the difference between the feed offered and orts on a DM basis.

During the same period of sampling feeds, 4 h after the morning feeding, blood samples were also collected via the coccygeal vein and artery to analyze the glucose, total protein, blood urea nitrogen, and insulin-like growth factor 1. Blood analyses were performed in a commercial laboratory using colorimetric-enzymatic, biuret, and fixed-time kinetic methods with an automated biochemical analyzer (BS200E, Mindray, Shenzhen, China) and commercial kits (Bioclin^®^, Belo Horizonte, Brazil). Insulin-like growth factor 1 was analyzed using an automated immunoassay analyzer (Immulite 2000, Siemens Healthcare Diagnostics, Erlangen, Germany).

From day 18 to 23, a cobalt-EDTA complex infusion (Co-EDTA) was carried out using an infusion pump, with 6 g cobalt in 4 L distilled water being infused into the rumen of each cow every 24 h. From day 21 to 23, eight samples of feces, urine, rumen content, and omasum content were taken at 9 h intervals (9 a.m. and 6 p.m. on the first day, 3 a.m., 12 p.m. and 9 p.m. on the second day, and 6 a.m., 3 p.m. and 12 p.m. on the third day).

Fecal samples were collected directly from the rectal ampulla. After collection, the samples were dried for 72 h at 55 °C in a forced-air oven and composited per cow across the eight sampling events. The composite samples were then ground to pass through 2 and 1 mm screens. The 1 mm samples were used for the determination of DM, OM, CP, and NDF, while the 2 mm samples were used for the determination of iNDF. The iNDF was used as an internal marker to estimate fecal DM excretion, which was calculated by dividing the ingested iNDF DM by the percentage of iNDF in the feces.

To calculate the microbial protein synthesis and microbial efficiency, and to evaluate the nitrogen balance and excretion, spot urine samples were taken. The collected volume was filtered through gauze and stored in two aliquots. The first aliquot consisted of 50 mL of pure urine, while the second aliquot was formed from 10 mL of pure urine and 40 mL of sulfuric acid (0.036 N). For the analysis of creatinine, urea, uric acid and allantoin, pool samples were prepared from the eight collections, using the same amount of each sample. The concentrations of creatinine, uric acid, and urea were quantified in diluted urine by the colorimetric kinetic method, the colorimetric-enzymatic method and the fixed-time kinetic method, respectively, using an automated biochemical analyzer (BS200E, Mindray, Shenzhen, China) and commercial kits (Bioclin^®^, Belo Horizonte, Brazil). The allantoin concentration in the urine was determined according to the method described by Chen & Gomes [29]. The urine volume (L) was calculated using the average creatinine excretion rate of 29 (mg/kg), using the following equation: body weight (kg) × 29/creatinine concentration (mg/L) [30,31]. Microbial protein synthesis was calculated according to the method described by Chen & Gomes [29], where the excretion of purine derivatives (*PD*, mmol/d) was calculated as the sum of the amounts of allantoin and uric acid excreted in urine. The absorbed purines (*AP*, mmol/d) were calculated according to the following equation:

(2) $$AP=\;\frac{\left(PD-(0.385\times \left({BW}^{0.75}\right))\right)}{0.85}$$

where total *PD* = excretion of purine derivatives (mmol/d); 0.385 = endogenous contribution to purine excretion; *BW*^0.75^ = metabolic body weight of the animal (kg); and 0.85 = recovery of absorbed purines as purine derivatives in urine. The rumen synthesis of microbial compounds (*Nmic*, g/d) was calculated according to the following equation:

(3) $$Nmic=\;\frac{AP\;\times \;70}{(0.116\times 0.83\times 1000)\;}$$

where *AP* = absorbed purines (mmol/d), 70 = nitrogen content of microbial purines (mg, N/mmol); 0.116 = the ratio of purine-N/total N of bacteria; and 0.83 = intestinal digestibility of microbial purines.

The production of true digestible microbial protein (*TPmicd*, g/d) was calculated using the following equation:

(4) TPmicd=Nmic×6.25×0.659

where *Nmic* = ruminal synthesis of microbial compounds (g/d) and 0.659 = conversion factor for microbial CP, considering that microbial CP consists of 82.4% true protein and is 80% digestible [27].

Microbial efficiency was calculated as the ratio between microbial protein synthesis and the intake of digestible OM. Nitrogen balance was calculated as the difference between the total nitrogen ingested and the total nitrogen excreted in feces, urine, and milk.

The rumen contents were sampled to determine the rumen pH, rumen ammonia nitrogen (RAN) and VFA profile. The rumen content collected at the liquid–solids interface was filtered through a nylon filter with a porosity of 100 µm and the pH was measured using a digital pH meter (Tecnal Equipamentos Científicos, model Tec-3MP pH meter [M14.1], Piracicaba, SP, Brazil). An aliquot of 40 mL was fixed with 1 mL sulfuric acid (1:1) and stored at −15°C for analysis of the RAN concentration according to the method described by Chaney & Marbach [32]. A second aliquot of 20 mL was fixed with 5 mL metaphosphoric acid (25% *w*/*v*) and stored at −15°C for evaluation of the VFA profile, which was analyzed by high-performance liquid chromatography. Samples were treated according to the method described by Siergfried et al. [33] with a Shimadzu LC20AT chromatograph (LC-20AT, Shimadzu, Kyoto, Japan) coupled to an RID-20A refractive index detector (RID-20A, Shimadzu, Kyoto, Japan), using sulfuric acid (H_2_SO_4_) at a concentration of 5 mmol/L and a flow rate of 0.7 mL/min.

The omasal contents were collected and sampled to determine the content flux using the technique described by Huhtanen et al. [34] and adapted by Leão [35]. Approximately 1 L of the sample was filtered through 100 µm nylon filters with a pore area of 44% surface area (Sefar Nitex 100/44, Sefar, Thal, Switzerland) and divided into two phases: a liquid phase with small particles, which was filtered, and a solid phase with large particles, which remained on the filter. The samples were freeze-dried and grounded in a 2 and 1 mm knife mill and composite samples of each animal were prepared for the eight collections performed in each period. The content flux was estimated using a dual-marker system, with cobalt as a marker for the liquid phase and iNDF as a marker for the solid phase [36,37]. The cobalt concentration in the different phases of omasal digesta was analyzed by inductively coupled plasma atomic emission spectrometry (Optima, PerkinElmer, Waltham, MA, USA) and calculated using the M-005/2 method according to Detmann et al. [28].

On day 24, 4 h after offering the diet, the rumen of the fistulated animals was completely emptied to assess the passage rate and rumen degradation, using the technique described by Allen & Linton [38]. The contents were filtered to separate the liquid and solid portions and stored separately in 65 L plastic buckets. At the end of rumen content emptying, the portions were weighed, samples were taken and the digesta was reconstituted and returned to the rumen of each animal. On day 26, the same procedure was carried out, but before the first feeding of the day. The samples were dried in a forced-air oven at 55 °C for 72 h, freeze-dried and grounded in a knife mill through 1 and 2 mm sieves. The rumen pool was calculated by averaging the rumen pool measured on days 24 and 26 by rumen emptying. Ingestion, passage and digestion rates were determined as suggested by Allen & Linton [38]. The digestion rate (kd; %/h) was calculated from the difference between the ingestion rate (ki; %/h) and the passage rate (kp; %/h). The ingestion and passage rates were calculated using the following equations:

(5) $$ki=\left(\frac{Intake}{Rumen\;pool}\right)\times 100$$

where ki = ingestion rate (%/h), intake (kg/h) and rumen pool (kg).

(6) $$kp=\left(\frac{Rumen\;outflow}{Rumen\;pool}\right)\times 100$$

where kp = passage rate (%/h), rumen outflow (kg/h) and rumen pool (kg).

Between days 24 and 28, the CH_4_ emitted by the animals was measured using the sulfur hexafluoride (SF_6_) tracer gas technique, as described by Primavesi et al. [39]. Two days prior to the start of the 5 day CH_4_ measurement period, two permeation capsules with a known SF_6_ emission rate were inserted into the rumen of the non-fistulated animals via an oro-oesophageal probe. On day 24, each animal was equipped with a halter, a capillary tube system and an evacuated PVC yoke connected to a vacuum pump, with the initial pressure recorded. An additional evacuated yoke fitted with a capillary tube was positioned in the environment to measure the ambient CH_4_ and SF_6_ concentrations. After 24 h of collection, the yokes were replaced with new evacuated ones. The pressure of the collected yokes was recorded, and they were pressurized by dilution with nitrogen, followed by a second measurement. Ruminal gas was withdrawn from each yoke using a disposable syringe and needle and transferred into 30 mL evacuated penicillin vials with rubber stoppers. These vials had been previously evacuated using a manual vacuum pump, with five sample vials per yoke, per animal and per day. The environmental yoke was collected simultaneously with those from the animals and subjected in the same manner for storage.

The concentration of CH_4_ in the yoke was determined by gas chromatography with a flame ionization detector. The concentration of SF_6_ in the yoke was determined using a gas chromatograph equipped with an electron capture detector. From the concentrations of CH_4_ and SF_6_ measured in the yoke and the known emission rate of SF_6_, the CH_4_ emission rate of the animal was calculated using the following equation:

(7) $$E_{CH4}=\left\{\frac{\left[A\text{\_}CH_{4}]-[B\text{\_}H_{4}\right]}{\left[A\text{\_}SF_{6}\right]}\right\}\times 60$$

where E_CH4_ = CH_4_ emission rate (g/h); E_SF_6_ = SF_6_ emission rate through the permeation capsule (g/min); A_CH_4_ = CH_4_ concentration in the yoke of the animals (µg/m^3^); B_CH_4_ = CH_4_ concentration in the environment (µg/m^3^); and A_SF_6_ = SF_6_ concentration in the yoke of the animals (µg/m^3^).

### 2.3. Statistical Analyses 

The data were analyzed using the lmer function of the lme4 package of R, according to a replicated Latin Square design as follows:

(8) $$Y_{ijklm}=\mu +T_{i}+{QL}_{j}+A_{\left(j\right)k}+P_{\left(j\right)l}+\;\varepsilon_{ijklm}$$

where Y_ijklm_ = dependent variable; μ = overall mean; T*_i_* = fixed effect of treatment; QL*_j_* = random effect of Latin Square; A*_(j)k_* = random effect of animal within Latin Square; P*_(j)l_* = random effect of period within Latin Square, and ε*_ijklm_* = random error. The interaction between treatment and Latin Square was not significant for all variables and was removed from the statistical model.

After the analysis of variance, the treatments were subjected to Tukey’s test for mean comparisons. The model assumptions were verified by evaluating the residual normality and homogeneity of variance prior to the interpretation of the results. For all analyses, 0.05 was considered the significant level for type I error and *p*-values between 0.05 and 0.10 were considered a trend.

## 3. Results

### 3.1. Intake and Digestibility

The NDF intake was higher in animals in the CON and SUP treatments than in the MON treatment (*p* = 0.029; Table 2). The intake of DM, OM and CP did not differ among treatments (*p* > 0.05). The apparent total tract digestibility of DM, OM, CP, and NDF did not differ among treatments (*p* > 0.05). The apparent degradability of NDF in the rumen was higher in the CON treatment (*p* = 0.008). The potentially digestible neutral detergent fiber (pdNDF) was also higher in the CON treatment (*p* = 0.011). The intestinal digestibility of DM, CP, NDF, and pdNDF did not differ among treatments (*p* > 0.05).

### 3.2. Milk Yield and Composition

Milk yield, ECM, and milk composition in terms of fat, protein, lactose, casein, MUN, and SCC did not differ among treatments (*p* > 0.05; Table 3).

### 3.3. Methane

Total CH_4_ emissions, CH_4_ emission per liter of milk yield and CH_4_ emission per liter of ECM did not differ among treatments (*p* > 0.05; Table 4). The CH_4_ emissions per kg of DMI, CH_4_ emission per unit of organic matter intake and CH_4_ emission per unit of digestible organic matter intake tended to differ among treatments (*p* = 0.091, *p* = 0.093, *p* = 0.086).

### 3.4. Rumen Parameters

**Figure 1:**
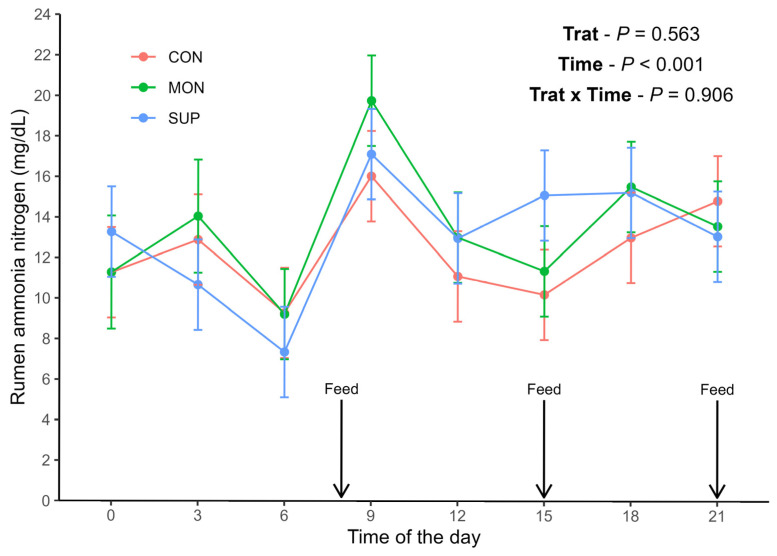
Concentration of RAN (mg/dL) of Holstein cows at different times of the day when fed diets without feed additives (CON), with sodium monensin (MON) or with a supplement containing *Acacia* tannins plus yeast products (SUP).

The total production of VFA and the proportions of acetate, propionate, and butyrate relative to total VFA were not affected by treatments (*p* > 0.05; Table 5). The mean RAN concentrations were 12.3, 13.5, and 13.1 mg/dL for CON, MON, and SUP, respectively (Figure 1). No treatment effect (*p* = 0.563) or treatment × time interaction (*p* = 0.906) was observed for RAN.

A significant effect of time was detected (*p* < 0.001), with the lowest mean RAN at time 6 (8.62 mg/dL) and the highest at time 9 (17.64 mg/dL) (Figure 1).

**Figure 2:**
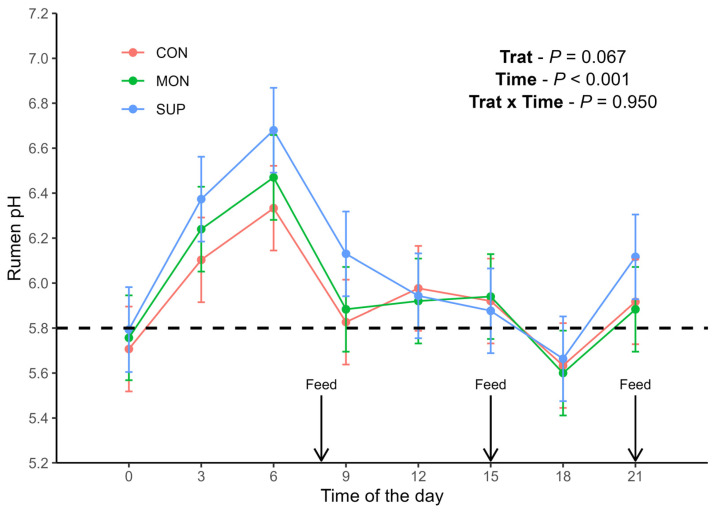
Average rumen pH of Holstein cows at different times of the day when fed diets without feed additives (CON), with sodium monensin (MON) or with a supplement containing *Acacia* tannins plus yeast products (SUP). The dashed line represents the threshold pH of 5.8.

Rumen pH tended to differ among treatments (*p* = 0.067, Figure 2). The average rumen pH was 5.93 for CON, 5.96 for MON and 6.07 for SUP. The standard error was 0.147. An effect of time was observed (*p* < 0.001): the lowest mean rumen pH was observed at time 18 (pH = 5.63) and the highest mean rumen pH was found at time 6 (pH = 6.49). 

### 3.5. Serum Blood Parameters

The animals in the CON, MON, and SUP treatments showed similar values for glucose, blood urea nitrogen (BUN), and total protein (*p* > 0.05, Table 6).

### 3.6. Nitrogen Balance and Microbial Protein

Urea excretion, microbial protein synthesis, Rumen undegradable protein (RUP) flow, and microbial efficiency did not differ among treatments (*p* > 0.05; Table 7). The nitrogen intake was similar in all treatments, as was N excretion in feces, urine and milk. The N balance therefore did not differ among treatments.

### 3.7. Rumen Kinetics and Characteristics

Table 8 summarizes the rumen pool size, ingestion rate, passage rate, and digestion rate across treatments.

Overall, most rumen kinetic parameters were not affected by treatments. However, the rumen CP pool differed among treatments (*p* = 0.014), and was lower in SUP. In addition, the NDF rumen pool tended to differ among treatments (*p* = 0.093).

Regarding ingestion kinetics, SUP increased the ingestion rates of DM (*p* = 0.049), CP (*p* = 0.028), and NDF (*p* = 0.013) compared with CON and MON.

No treatment effects were observed for passage rate variables (*p* > 0.05), although the passage rate of iNDF tended to differ among treatments (*p* = 0.090).

For digestion kinetics, the pdNDF digestion rate differed among treatments (*p* = 0.007), and was lower in MON than in CON and SUP.

There was a difference among CON, MON, and SUP treatments for the ingestion rate of DM (*p* = 0.049), CP (*p* = 0.028) and NDF (*p* = 0.013), with the ingestion rate being higher in the animals in the SUP treatment than in the animals in the CON and MON treatments. The passage rate of iNDF tended to differ among CON, MON and SUP treatments (*p* = 0.090), with the passage rate of iNDF being higher in the SUP treatment than in the CON treatment, and there was no difference between the MON treatment and the CON treatment of the SUP treatment. The pdNDF digestion rate differed on average between the CON, MON and SUP treatments (*p* = 0.007), with the pdNDF digestion rate being higher in the CON and SUP treatments than in the MON treatment, and there was no difference between the CON and SUP treatments.

## 4. Discussion

The results of our study showed no differences in DMI among treatments. Reductions in DMI have been observed when the animals are fed high doses of monensin, with the magnitude of the reduction increasing as the dose increases [14]. Significant decreases in DMI, milk protein, milk fat content, and milk fat yield have been observed when the doses used were of 18 to 50 mg/kg [13,40]. Since the dose of monensin offered in the present study was 12 mg/kg DM, this could explain the lack of reduction in intake. The meta-analysis conducted by Berça et al. [24] observed no reduction in DMI in response to tannin supplementation at doses below 124 g/kg DM, which supports our findings. The decrease in intake associated with tannin supplementation is related to palatability; however, this effect usually occurs when high doses of tannins are used [41]. Moreover, the effect of tannins depends not only on the amount ingested, but also on the type of tannins, their chemical structure and molecular weight [42].

The results for OM and CP intakes differed from those reported by Silva et al. [43], where the OM and CP intakes were higher in the control treatment (OM = 11.5 kg/d; CP = 1.84 kg/d) compared with the monensin treatment (OM = 9.63 kg/d; CP = 1.52 kg/d). In the present study, they did not differ among treatments. Additionally, the apparent total tract digestibility, rumen degradability and intestinal digestibility of CP did not differ. Nevertheless, the rumen pool of CP was lower in the SUP treatment.

These results may be associated with differences in intake and digestion rates, which tended to be higher, although not significantly. Therefore, the responses should be interpreted with caution. Tannins have the ability to form insoluble complexes with proteins in the rumen, increasing their passage rate to the intestine [12,44]. However, this mechanism was not sufficient to affect nitrogen balance and microbial protein synthesis.

The NDF intake was higher in the SUP treatment compared to the MON treatment. Costa et al. [45] observed an increase in NDF intake in lambs supplemented with condensed tannins, which supports the results of our study. However, Nascimento et al. [46] observed no effect on NDF intake in goats supplemented with tannins. The ruminal degradability of NDF and pdNDF was lower in the MON treatment, which was likely due to the inhibitory effect on Gram-positive bacteria that degrade dietary fiber [47]. Similarly, reductions in NDF and pdNDF’s ruminal degradability observed in the SUP treatment may be associated with the presence of tannins, as they can reduce the digestion of dietary fiber by inhibiting cellulolytic bacteria or fibrolytic enzymes [42].

On the other hand, yeasts can stimulate the growth of cellulolytic bacteria [25]. In the present study, the higher NDF ingestion rate observed in the SUP treatment was associated with a tendency for an increased iNDF passage rate. In addition, the NDF digestion rate tended to be higher, with a significantly higher digestion rate observed for pdNDF. This could therefore explain the fact that the rumen pool was smaller in the SUP treatment, suggesting a dynamic balance between the intake, passage, and digestion rates.

In terms of milk yield, Ahvanooei et al. [14] observed an increase when animals were supplemented with up to 23 ppm of monensin, with no significant effect for supplementation from 24 ppm to 38 ppm, and a decrease when animals received doses above 38 ppm. These results differ from those found in our study. Aguerre et al. [48] and Menci et al. [44] observed no effect on milk production in animals supplemented with chestnut (*Castanea sativa*) and quebracho (*Schinopsis lorentzi*) tannins. In contrast, Abdelli et al. [49] reported in a multilevel meta-analysis and meta-regression increased milk yield associated with the supplementation of yeast products.

Regarding tannins, in a meta-analysis, Herremans et al. [50] reported a significant 1.7% increase in milk yield, whereas in our study, the milk yield increased by 3.8% in the SUP treatment compared to the CON treatment, although this difference was not statistically significant. The statistical significance observed in the meta-analysis likely reflects its greater statistical power due to the inclusion of a larger number of observations across studies. In contrast, the relatively small sample size in the present experiment may have limited the statistical power to detect treatment effects, even when numerical differences were observed. Therefore, the results should be interpreted with caution, and future studies with a greater number of experimental units are needed to confirm these responses.

According to Ahvanooei et al. [14], the increase in milk yield as well as lactose is due to higher glucose availability resulting from increased propionate production and decreased amino acid deamination in the rumen. In the present study, no difference was found in propionate production and glucose levels. However, in our study, tannins were supplemented together with yeast products. According to Petri et al. [51], yeast extracts may stimulate the expression of transporters in the rumen epithelium, enhancing VFA absorption and increasing the postruminal supply of energy, which might explain the higher yield of the SUP treatment.

Ahvanooei et al. [14] observed a decrease in the milk fat percentage when animals were supplemented with up to 51 ppm of monensin with no effects observed outside the range of 21 ppm to 31 ppm. In the present study, monensin was provided at 12 ppm, which is below this range. Monensin can reduce milk fat synthesis by inhibiting the bacteria involved in ruminal biohydrogenation pathways and decreasing acetate production, thereby limiting the availability of key precursors for de novo milk fat synthesis [52]. In contrast, the absence of a reduction in milk fat can be attributed to propionate as a carbon source for fatty acids elongation, and an increase in intestinal AA flux, possibly stimulating de novo synthesis via mTOR signaling [52]. Moreover, Piantoni et al. [53], Aguerre et al. [48], Herremans et al. [50] and Menci et al. [54] found no effects of tannins on the milk fat content, which is consistent with the results of our study.

Overall, our findings align with the majority of studies reporting limited or inconsistent effects of monensin and tannins on milk protein and lactose content. Despite previous reports of reduced milk protein at monensin doses ranging from 12 to 36 ppm [14], in the MON treatment that consisted of 12 ppm of monensin, no reduction was observed. This result may be related to the similar milk yield across treatments [52,53]. Regarding tannins, Herremans et al. [50] and Menci et al. [54] observed no effect on the milk protein, while Aguerre et al. [48] and Grazziotin et al. [41] observed an increase. As for lactose, the addition of monensin at doses of 16 to 96 ppm increases the amount of lactose in milk [14], a result that differs from those observed in this study. Similarly, Horst et al. [52] and Aguerre et al. [48] reported no effect of tannin intake on the lactose content, which is consistent with the results of our study.

Energy-corrected milk (ECM) did not differ among treatments, which is consistent with the results of Piantoni et al. [53] for monensin supplementation and Herremans et al. [50] for tannin supplementation. In contrast, Grazziotin et al. [41] observed an increase in ECM in animals supplemented with tannins, due to increased milk production. Horst et al. [52] also found a higher ECM in animals supplemented with monensin.

Regarding MUN, an increase was observed when the animals were fed doses of monensin between 13 and 30 ppm, while the other doses showed no effect [14]. This result is attributed to lower microbial degradation and an increased flow of undegradable protein from the rumen to the intestine. This results in a greater contribution of absorbed AA from the intestine to the AA profile of the milk, which is confirmed by the lower ammonia levels observed in the rumen [14]. Similarly to the present study, Horst et al. [52] and Piantoni et al. [53] observed no effects on monensin supplementation. Regarding tannins, Herremans et al. [50] observed an 8% reduction in MUN due to lower protein degradability in the rumen and reduced ammonia formation, as did Menci et al. [54], which differ from the results of our study.

Although some positive (non-significant) responses were observed in milk and in methane-related variables, as will be discussed in further sections, the use of both additives did not translate into improvements in feed efficiency. Specifically, no statistical differences were detected for feed efficiency when expressed as milk yield per unit of DMI or as energy-corrected milk per unit of DMI. Therefore, the treatments did not significantly improve the efficiency of converting the feed intake into the milk output.

From an applied perspective, these results suggest that the potential benefits of the additive in terms of methane mitigation and ruminal responses should be interpreted cautiously, as they were not accompanied by improvements in productive efficiency. Since the feed efficiency is a key determinant of economic returns in dairy systems, the absence of significant changes in milk output relative to feed intake indicates that the economic viability of the additive may be limited under the conditions evaluated. Consequently, further studies are needed to assess whether the observed responses could translate into consistent productive or economic benefits under different production scenarios.

As for emission variables, the total CH_4_ emission (g/d) did not differ among treatments, as also observed in the meta-analysis by Ahvanooei et al. [14] for monensin, Brutti et al. [55] for tannins, and Darabighane et al. [56] for yeasts. However, it differed from the findings of Xue et al. [57], Roca-Fernández et al. [16] and Muñoz et al. [58] for monensin, tannins and yeast, respectively.

CH_4_ emission per kg DMI tended to be lower in the treatments receiving additives, with an 8.54% decrease for MON and 17.25% decrease for SUP when both are compared to the CON treatment. Similarly to our study, Battelli et al. [17] reported a 17.8% statistically significant decrease in CH_4_ emission per kg DMI when goats were supplemented with quebracho condensed tannin. Such responses in emissions per Kg of DMI could be related to the lower DMI rate in the SUP treatment. In our study, CH_4_ emission per kg OMId also tended to be lower, a 16.8% of reduction for SUP and 7.9% for MON, a decrease also reported by Battelli et al. [17] (7.6%). Finally, CH_4_ emission per kg OMI also tended to be lower in the SUP treatment than in the MON and CON treatment (8.8% and 17.6% of difference, respectively), with the results being consistent with those from the literature about additive-fed animals [16]. Despite the decrease in CH_4_ emission per kg DMI, OMI and OMId, in a similar manner to that reported in the literature when animals are fed tannins, the results found in our study are tendencies towards significance and are not statistically significant.

Lower CH_4_ production was expected in animals fed with additives (MON or SUP) due to the lower apparent ruminal degradability of NDF and pdNDF observed for these treatments, which would reduce the formation of H_2_, acetate and methanogenic microorganisms [24]. This lower degradability could also explain the tendency to a lower CH_4_ production per kg of digestible OM in the SUP treatment compared to the CON treatment, as observed by Roca-Fernández et al. [16] and Battelli et al. [17]. Across studies, bioactive compounds derived from plants are reported to modulate rumen fermentation pathways and host immune responses, thereby altering microbial activity and metabolic hydrogen utilization [59]. Such effects may help to explain the variability in CH_4_ emissions and animal performance responses when these additives are included in the diet.

The rumen pH was similar between the treatments receiving any type of feed supplement and the CON treatment. However, the animals in the SUP treatment exhibited a higher rumen pH than those in the CON treatment. Higher pH values can be expected when animals receive condensed tannins due to their buffering capacity [16] or to a influence they may play on chewing and rumination, increasing salivation and controlling rumen pH [42]. In addition, yeasts may contribute to rumen pH stabilization by stimulating the proliferation of bacteria that utilize lactate and by reducing the activities of lactate-producing microbes [25]. Nevertheless, a limitation of the present study is that the rumen microbiota composition was not evaluated, which precludes firm conclusions regarding microbial shifts underlying the observed differences in rumen pH. Thus, future studies evaluating the rumen microbiota in response to these treatments could provide further answers to the mechanisms involved and to explain the results found.

Regarding the total production of VFA, similarly to a study by Piantoni et al. [53], we observed no difference in the total VFA. However, the same study [53] reported a decrease in acetate and butyrate production and an increase in propionate production in animals supplemented with monensin. This result is expected because of monensin in inhibiting Gram-positive bacteria and the resulting proliferation of Gram-negative bacteria. However, these findings diverge from those found in our study, as the percentage of propionate, acetate and butyrate in relation to the total VFA did not differ among treatments.

Berça et al. [24] observed an increase in the total VFA as well as propionate and butyrate and found no effect on acetate when tannins were used. Tannins stimulate the synthesis of propionate, a H_2_ sink, while butyrate and acetate form H_2_. In addition, protozoa and methanogenic archaea have a synergistic interaction that facilitates the transfer of H_2_ to the methanogens. Therefore, increased propionate production can lead to lower CH_4_ production, using H_2_ that would otherwise be used for methanogenesis [24]. On the other hand, Battelli et al. [17] found no difference in the total production of VFA in goats fed with quebracho (*Schinopsis balansae*) tannins.

The concentration of RAN observed in our study was similar among treatments, which is consistent with the meta-analysis by Ahvanooei et al. [14]. According to Ahvanooei et al. [14], factors such as DMI, the nutrient composition of the feed, monensin dosage and the duration of supplementation may influence the results. This could explain the different results in the literature regarding RAN concentration. Piantoni et al. [53] observed that monensin reduced the RAN compared to the control treatment, while Silva et al. [43] found an increase in the RAN concentration.

Regarding tannins, Herremans et al. [50] observed a reduction in RAN when the animals were supplemented with tannins, which was attributed to lower protein digestibility in the rumen and increased protein passage into the intestine. As no change in protein digestibility was observed in their study, this could explain the similar concentrations of RAN found. Similarly, Chung et al. [60] compared active dried *Saccharomyces cerevisiae* strains and reported that RAN was similar among treatments. This result diverges from those reported by Takiya et al. [61] and Hirstov et al. [62], in which yeast supplements decreased the RAN concentrations.

As for the blood parameters, the glucose levels were similar among the treatments assessed, which is consistent with the meta-analysis by Ahvanooei et al. [14] for monensin supplementation, Grazziotin et al. [41] for the use of tannins and Takiya et al. [61] for the use of yeasts. Approximately 27% to 54% of glucose synthesized in the liver comes from propionate [63]. As propionate production did not differ among treatments, this could explain the similar results for the glucose levels.

Blood urea nitrogen was also similar among treatments, in agreement with Ahvanooei et al. [14] for monensin supplementation, which is consistent with the results found in our study. Results are also consistent with the study by Takya et al. [61] that did not observe differences when using yeasts. However, it differs from Grazziotin et al. [41], who observed a trend towards increased BUN, and Herremans et al. [50], who observed a 9% reduction in BUN with tannin supplementation. The breakdown of CP in the rumen produces peptides, AA and RAN. Excess ammonia from the rumen is absorbed into the bloodstream and converted to urea in the liver. This urea is then excreted in the urine, milk or blood or returned to the rumen through urea recycling [41]. As no significant effects on CP degradation and RAN were observed, this could explain the lack of an effect on BUN. Additionally, the total blood protein did not differ among the treatments evaluated, which is consistent with the result found by Silva et al. [43] for monensin supplementation and Battelli et al. [17] for dairy goats with *Schinopsis balansae* tannins supplementation. 

Condensed tannins can form complexes with proteins, potentially reducing the availability of nutrients for microbial protein synthesis in the rumen, and increasing the availability of postruminal protein [44,64]. However, consistent with the literature, the microbial protein synthesis, microbial protein synthesis efficiency and RUP flow did not differ among treatments [19,42,43,44,65,66].

The nitrogen intake did not differ among treatments, which is consistent with the findings of Ahvanooei et al. [14] in a meta-analysis that found no association between nitrogen intake and monensin supplementation. Aguerre et al. [48] observed an increase in the nitrogen intake when animals were supplemented with tannins, but this increase is related to the increase in DMI, which was not the case in our study.

Urinary nitrogen excretion also did not differ among treatments, similar to the observations of Ahvanooei et al. [14] in animals supplemented with monensin and Renno et al. [66] for low levels of *Acacia mearnsii* tannins. However, it differed from the results of Herremans et al. [50] and Tseu et al. [42], in which urinary nitrogen excretion was lower when animals were supplemented with tannins, due to lower protein degradability, lower ammonia nitrogen production in the rumen and lower urea formation in the liver.

Fecal nitrogen was reduced by the addition of monensin at a dosage of 14 to 22 mg/kg, as reported by Ahvanooei et al. [14] and by Tseu et al. [42]. In the present study, monensin was supplied at 12 mg/kg, which is slightly below this range, and no differences were observed. However, it was not affected by the addition of tannins [65] and yeasts [61] which is consistent with the results of our study. Nitrogen balance did not differ between treatments, as the nitrogen intake and excretion in urine, feces and milk were similar. These results can be explained by the lack of differences in crude protein digestibility between the treatments studied [43].

From a broader perspective, these findings suggest that the evaluated additives maintained nitrogen utilization efficiency without increasing nitrogen losses to the environment. Future research should explore a wider range of inclusion rates and longer supplementation periods to determine whether more pronounced shifts in nitrogen partitioning can be achieved.

In addition, integrating rumen microbial profiling and isotopic or metabolomic approaches could help to clarify the mechanisms regulating ruminal dynamics, nutrient transformations, absorption, and excretion pathways. Such evaluations would contribute to the development and use of plant-derived compounds in feeding strategies that lower CH_4_ emissions and optimize production efficiency while minimizing environmental nitrogen losses in dairy systems. The economic feasibility, cost benefit ratio, and significantly long-term sustainability of using such additives under commercial conditions remain uncertain and were beyond the scope of the present study, while also being an interesting point to be investigated.

## 5. Conclusions

The addition of a blend of tannin and yeast in the diet of lactating Holstein dairy cows tended to reduce CH_4_ emissions relative to DMI, OMI and digestible OM when compared to sodium monensin. However, both tested additives did not reduce the total CH_4_ emissions. The milk yield and DM intake also did not change significantly when using tannins and yeasts. Thus, despite numerical responses observed in the yield, no improvements were observed in the feed efficiency when expressed as the milk yield or energy-corrected milk per unit of dry matter intake. From a practical perspective, the findings suggest that although the tannin and yeast blend may contribute to reductions in the methane yield, its use did not improve productive efficiency under the conditions of the present study. Further research is required to better understand the mechanisms of action involved, particularly the effects of tannins and yeasts on the rumen microbiome, long-term effects and their potential to mitigate methane emissions in dairy production systems under different circumstances.

## Figures and Tables

**Table 1:** Experimental diet composition used for Holstein cows receiving diets with different types of feed additives.

Ingredient, g/kg	Treatments ^1^		
	CON	MON	SUP
Corn silage	560	560	560
Tifton hay	40	40	40
Soybean meal	151	151	150
Corn grain finely ground	122	122	121
DDG ^2^	99	99	99
Dicalcium phosphate	8	8	8
Urea	8	8	8
Commercial buffer ^3^	4	4	4
Salt	3.9	3.9	3.9
Limestone	2	2	2
Sulfur flower	0.9	0.9	0.9
Mineral premix ^4^	0.5	0.5	0.5
Mycotoxin adsorbent	0.4	0.4	0.4
Virginamycin	0.4	0.4	0.4
Sodium monensin	-	0.012	-
Supplement ^5^	-	-	2
Diet composition, g/kg			
Dry matter	569	569	568
Organic matter	949	948	949
Crude protein	177	174	173
RDP ^6^	119	119	120
Starch	275	274	273
Fat	44	44	44
Neutral detergent fiber	379	362	345

^1^ CON = control, MON = monensin, SUP = supplement containing Acacia tannins and yeast products; ^2^ DDG = dried distillery grains; ^3^ buffer composed of sodium bicarbonate, calcareous seaweed and magnesium oxide; ^4^ calcium: 40 g/kg, cobalt: 280 mg/kg, copper: 11 g/kg, sulfur: 200 mg/kg, iodine: 600 mg/kg, magnesium: 150 g/kg, manganese: 25 g/kg, selenium: 250 mg/kg, zinc: 40 g/kg, vitamin A: 4,000,000 UI/kg, vitamin D: 1,000,000 UI/kg, vitamin E: 25,000 UI/kg; ^5^ chemical composition of the supplement based on condensed Acacia tannins and *Saccharomyces cerevisiae* (SUP)—DM: 88.9%, OM: 49.6%; CP: 17.2%; NDFa: 18.0%; and ^6^ rumen degradable protein.

**Table 2:** Intake and apparent digestibility in Holstein cows fed diets with different types of feed additives.

Item	Treatments ^1^			SE ^2^	*p*-Value
	CON	MON	SUP		
Intake, kg/d					
DM ^3^	22.8	22.9	23.4	1.27	0.364
OM ^4^	21.4	21.4	22.0	1.19	0.366
CP ^5^	4.22	4.16	4.21	0.22	0.709
NDF ^6^	8.70 a	8.26 b	8.81 a	0.491	0.029
Apparent total tract digestibility, g/kg					
DM	646	639	634	14.9	0.644
OM	668	659	657	14.6	0.601
CP	721	720	709	14.4	0.486
NDF	540	509	517	23.9	0.177
Apparent ruminal degradability, g/kg of intake					
DM	614	586	567	31.5	0.117
CP	618	556	558	44.3	0.131
NDF	423 a	294 b	333 b	55.6	0.008
pdNDF ^7^	544 a	388 b	430 b	70.8	0.011
Intestinal digestibility, g/kg of rumen outflow					
DM	159	139	142	44.0	0.863
CP	275	371	365	72.6	0.472
NDF	225	301	291	42.7	0.112
pdNDF	453	564	492	79.7	0.343

^1^ CON = control, MON = monensin, SUP = supplement containing tannins and yeast products; ^2^ standard error; ^3^ DM = dry matter; ^4^ OM = organic matter; ^5^ CP = crude protein; ^6^ NDF = neutral detergent fiber; ^7^ pdNDF = potentially digestible neutral detergent fiber. Values within a row with different letter differ significantly, *p* < 0.05.

**Table 3:** Milk yield and composition in Holstein cows fed diets with different types of feed additives.

Item	Treatments ^1^			SE ^2^	*p*-Value
	CON	MON	SUP		
Milk yield, kg/d	36.7	38.3	38.1	2.06	0.323
ECM ^3^, kg/d	33.5	35.6	35.0	1.93	0.301
Milk composition					
Fat, %	3.42	3.62	3.52	0.185	0.403
Fat, kg/d	1.26	1.37	1.33	0.093	0.316
Protein, %	3.17	3.14	3.14	0.042	0.372
Protein, kg/d	1.16	1.20	1.20	0.068	0.456
Lactose, %	4.90	4.85	4.85	0.025	0.386
Lactose, kg/d	1.80	1.86	1.85	0.104	0.514
Total milk solids, %	12.4	12.4	12.4	0.169	0.931
Casein%	2.55	2.54	2.54	0.054	0.935
MUN ^4^, mg/dL	21.0	20.9	20.2	0.584	0.239
SCC ^5^, log	30.4	32.0	41.4	12.40	0.189
Feed efficiency, kg/kg					
MY/DMI ^6^	1.63	1.69	1.63	0.067	0.120
ECM/DMI ^7^	1.49	1.58	1.48	0.075	0.131

^1^ CON = control, MON = monensin, SUP = supplement containing tannins and yeast products; ^2^ standard error; ^3^ energy-corrected milk yield; ^4^ milk urea nitrogen; ^5^ SCC = somatic cell count; ^6^ milk yield divided by dry matter intake; and ^7^ energy-corrected milk divided by dry matter intake.

**Table 4:** Methane emission in Holstein cows fed diets with different types of feed additives.

Item ^1^	Treatments ^2^			SE ^3^	*p*-Value
	CON	MON	SUP		
CH_4_, g/d	259	239	229	30.60	0.271
CH_4__MY, g/L	7.16	6.24	6.11	0.772	0.312
CH_4__ECM, g/L	7.73	6.49	6.59	0.761	0.263
CH_4__DMI, g/kg	11.71 a	10.71 ab	9.69 b	0.850	0.091
CH_4__OMI, g/kg	12.5 a	11.4 ab	10.3 b	0.908	0.093
CH_4__OMId, g/kg	19.0 a	17.5 ab	15.8 b	1.300	0.086

^1^ CH_4__MY = methane emission per unit of milk yield, CH_4__ECM = methane emission per unit of energy-corrected milk yield, CH_4__DMI = methane emission per unit of dry matter intake CH_4__OMI = methane emission per unit of organic matter intake, CH_4__OMId = methane emission per unit of digestible organic matter intake; ^2^ CON = control, MON = monensin, SUP = supplement containing tannins and yeast products; and ^3^ standard error. Values within a row with different letter differ significantly, *p* < 0.05.

**Table 5:** Production and profile of volatile fatty acids in Holstein cows fed diets with different types of feed additives.

Item ^1^	Treatments ^2^			SE ^3^	*p*-Value
	CON	MON	SUP		
Total VFA, mmol/L	27.8	26.5	28.4	3.46	0.900
Acetate, mmol/100 mmol	54.0	52.6	52.8	2.48	0.379
Propionate, mmol/100 mmol	34.8	36.2	34.9	2.88	0.311
Butyrate, mmol/100 mmol	11.1	11.2	12.2	0.59	0.169

^1^ Total VFA, mmol/L = total production of volatile fatty acids; Acetate, mmol/100 mmol = percentage of acetate in relation to the total production VFA; Propionate, mmol/100 mmol = percentage of propionate in relation to the total production VFA; Butyrate, mmol/100 mmol = percentage of butyrate in relation to total production VFA; ^2^ CON = control, MON = monensin, SUP = supplement containing tannins and yeast products; and ^3^ standard error.

**Table 6:** Serum concentrations in Holstein cows fed diets with different types of feed additives.

Item ^1^	Treatments ^2^			SE ^3^	*p*-Value
	CON	MON	SUP		
GLU, mg/dL	48.7	52.9	49.0	1.98	0.189
BUN, mg/dL	55.3	54.0	49.7	3.33	0.286
TP, g/dL	7.39	7.01	7.00	0.571	0.786
IGF-1, ng/mL	197	219	187	14.3	0.195

^1^ GLU = glucose, BUN = blood urea nitrogen; TP = total protein, and IGF-1 = insulin-like growth factor 1; ^2^ CON = control, MON = monensin, and SUP = supplement containing tannins and yeast products; and ^3^ standard error.

**Table 7:** Nitrogen balance and microbial protein in Holstein cows fed diets with different types of feed additives.

Item	Treatments ^1^			SE ^2^	*p*-Value
	CON	MON	SUP		
Nitrogen metabolism					
Urinary urea excretion, g/d	99.7	114.9	108.3	10.5	0.318
Microbial protein synthesis, g/d	3037	2817	3027	219	0.560
RUP ^3^ flow, g/d	1177	1337	1245	174	0.686
Microbial efficiency ^4^, g/kg	215	202	208	13.5	0.649
Nitrogen balance, g/d					
Intake	674	664	683	38	0.467
Fecal excretion	193	190	202	18.9	0.395
Urinary excretion	298	293	286	35.7	0.932
Milk secretion	193	186	191	11.9	0.885
Balance	−10.37	−4.23	4.19	25.92	0.912

^1^ CON = control, MON = monensin, SUP = supplement containing tannins and yeast products; ^2^ standard error; ^3^ RUP = rumen undegradable protein; and ^4^ microbial efficiency was calculated based on the relationship between microbial protein synthesis and digestible organic matter intake.

**Table 8:** Rumen kinetics, pool, intake rate, passage rate, and digestion rate in Holstein cows fed diets with different types of feed additives.

Item	Treatments ^1^			SE ^2^	*p*-Value
	CON	MON	SUP		
Rumen pool, kg					
Dry matter	12.1	12.4	10.9	0.94	0.220
Crude protein	2.15 ab	2.17 a	1.91 b	0.167	0.014
Neutral detergent fiber	5.30 a	5.36 a	4.78 b	0.426	0.093
iNDF ^3^	2.22	2.08	1.66	0.248	0.269
pdNDF ^4^	3.08	3.27	3.12	0.331	0.670
Rumen pool, g/kg of body weight					
iNDF	3.31	3.13	2.51	0.384	0.301
pdNDF	4.57	4.93	4.70	0.531	0.652
Ingestion rate, %/h					
Dry matter	8.08 b	8.03 b	9.54 a	1.08	0.049
Crude protein	8.48 b	8.40 b	9.60 a	0.089	0.028
Neutral detergent fiber	7.04 b	6.71 b	8.18 a	1.07	0.013
iNDF	3.84	4.27	5.80	1.05	0.120
pdNDF	9.50	8.31	9.46	1.10	0.336
Passage rate, %/h					
Dry matter	5.01	5.27	4.76	0.546	0.753
Crude protein	5.99	6.65	6.21	0.676	0.778
Neutral detergent fiber	2.62	3.16	2.63	0.322	0.450
iNDF	1.64 b	1.96 ab	2.25 a	0.335	0.090
pdNDF	2.49	4.07	2.80	0.576	0.218
Digestion rate, %/h					
Dry matter	3.07	2.77	4.78	1.393	0.210
Crude protein	2.49	1.75	3.39	1.364	0.494
Neutral detergent fiber	4.42 ab	3.55 b	5.55 a	1.242	0.105
pdNDF	7.01 a	4.24 b	6.66 a	1.341	0.007

^1^ CON = control, MON = monensin, SUP = supplement containing tannins and yeast products; ^2^ standard error; ^3^ iNDF = indigestible neutral detergent fiber; and ^4^ pdNDF = potentially digestible neutral detergent fiber. Values within a row with different letter differ significantly, *p* < 0.05.

## Data Availability

The data presented in this study are available upon request from the corresponding author.

## Abbreviations

The following abbreviations are used in this manuscript:

AA	Absorbed Amino Acids
AP	Absorbed Purines
SUP	A Mixture of Tannins and Yeast Products
BUN	Blood Urea Nitrogen
CH4	Methane
CH_4__DMI	Methane Emission Per Unit of Dry Matter Intake
CH_4__ECM	Methane Emission Per Unit of Energy-Corrected Milk Yield
CH_4__MY	Methane Emission Per Unit of Milk Yield
CH_4__OMI	Methane Emission Per Unit of Organic Matter Intake
CH_4__OMId	Methane Emission Per Unit of Digestible Organic Matter Intake
CO_2_	Carbon Dioxide
CON	Control
CP	Crude Protein
DM	Dry Matter
ECM	Energy-Corrected Milk
E_CH_4_	Methane Emission Rate
Gt	Gigatons
GLU	Glucose
H_2_SO_4_	Sulfuric Acid
ICP-AES	Inductively Coupled Plasma Atomic Emission Spectrometry
IGF-1	Insulin-Like Growth Factor 1
iNDF	Indigestible Neutral Detergent Fiber
ki	Ingestion Rate
kp	Passage Rate
MON	Monensin
MUN	Milk Urea Nitrogen
N_2_O	Nitrous Oxide
NDF	Neutral Detergent Fiber
Nmic	Rumen Synthesis of Microbial Compounds
pdNDF	Potentially Digestible Neutral Detergent Fiber
OM	Organic Matter
PD	Purine Derivatives
RAN	Rumen Ammonia Nitrogen
RUP	Rumen Undegradable Protein
SCC	Somatic Cell Count
SF_6_	Sulfur Hexafluoride
TP	Total Protein
TPmicd	True Digestible Microbial Protein
VFA	Volatile Fatty Acids
